# Validation of two prediction models of undiagnosed chronic kidney disease in mixed-ancestry South Africans

**DOI:** 10.1186/s12882-015-0093-6

**Published:** 2015-07-04

**Authors:** Amelie Mogueo, Justin B. Echouffo-Tcheugui, Tandi E. Matsha, Rajiv T. Erasmus, Andre P. Kengne

**Affiliations:** Non-Communicable Diseases Research Unit, South African Medical Research Council, Cape Town, South Africa; School of Health Sciences, Catholic University of Central Africa, Yaounde, Cameroon; Hubert Department of Global Health, Rollins School of Public Health, Emory University, Atlanta, GA USA; Department of Medicine, MedStar Health, Baltimore, MD USA; Faculty of Health and Wellness Science, Cape Peninsula University of Technology, Cape Town, South Africa; Division of Chemical Pathology, Faculty of Health Sciences, National Health Laboratory Service (NHLS) and University of Stellenbosch, Cape Town, South Africa; Department of Medicine, Groote Schuur Hospital, University of Cape Town, Cape Town, South Africa

**Keywords:** CKD, Discrimination, Calibration, Validation, Prediction model

## Abstract

**Background:**

Chronic kidney disease (CKD) is a global challenge. Risk models to predict prevalent undiagnosed CKD have been published. However, none was developed or validated in an African population. We validated the Korean and Thai CKD prediction model in mixed-ancestry South Africans.

**Methods:**

Discrimination and calibration were assessed overall and by major subgroups. CKD was defined as ‘estimated glomerular filtration rate (eGFR) <60 ml/min/1.73 m^2^’ or ‘any nephropathy’. eGFR was based on the 4-variable Modification of Diet in Renal Disease (MDRD) formula.

**Results:**

In all 902 participants (mean age 55 years) included, 259 (28.7 %) had prevalent undiagnosed CKD. C-statistics were 0.76 (95 % CI: 0.73–0.79) for ‘eGFR <60 ml/min/1.73 m^2^’ and 0.81 (0.78-0.84) for ‘any nephropathy’ for the Korean model; corresponding values for the Thai model were 0.80 (0.77-0.83) and 0.77 (0.74-0.81). Discrimination was better in men, older and normal weight individuals. The model underestimated CKD risk by 10 % to 13 % for the Thai and 9 % to 93 % for the Korean model. Intercept adjustment significantly improved the calibration with an expected/observed risk of ‘eGFR <60 ml/min/1.73 m^2^’ and ‘any nephropathy’ respectively of 0.98 (0.87-1.10) and 0.97 (0.86-1.09) for the Thai model; but resulted in an underestimation by 24 % with the Korean model. Results were broadly similar for CKD derived from the Chronic Kidney Disease Epidemiology Collaboration (CKD-EPI) formula.

**Conclusion:**

Asian prevalent CKD risk models had acceptable performances in mixed-ancestry South Africans. This highlights the potential importance of using existing models for risk CKD screening in developing countries.

**Electronic supplementary material:**

The online version of this article (doi:10.1186/s12882-015-0093-6) contains supplementary material, which is available to authorized users.

## Background

Chronic kidney disease (CKD) is increasingly common worldwide, especially in Sub-Saharan Africa [1]. CKD poses a real public health challenge related to its many complications including end-stage of renal disease (ESRD), cardiovascular diseases (CVD) and premature mortality [2]. Early stages of CKD are clinically silent, with many affected persons being detected at advanced stages, when expensive renal replacement therapies are inevitable [3]. In many developed countries, CKD detection in early stage has improved since the adoption of the Kidney Disease Outcomes Quality Initiative (KDOQI) [4] classification system. However, in most developing countries, especially Africa, uptake of screening remains a challenge [5, 6]. Indeed, the limited data on CKD prevalence in African countries reflects low disease awareness on this continent, due to the lack of routine testing of at-risk populations including those in the early stages of CKD [6].

Screening for CKD, followed by implementation of interventions or strategies to prevent or delay transitions to ESRD (requiring replacement therapy i.e., dialysis or kidney transplant) as well as cardiovascular complications can potentially reduce the disease burden and costs. Current recommendations suggest the screening of individuals at risk of CKD on the basis of risk factors such as age, sex, diabetes mellitus, hypertension, dyslipidemia, high-normal urinary albumin excretion, family history of kidney failure or concurrent cardiovascular disease (CVD), rather than to quantify the cumulative effect of several risk factors [3, 4]. Algorithms to assess the overall/global CKD risk by simultaneously incorporating various risk factors have been developed and tested, and may facilitate the care for CKD in routine clinical practice [7]. These risk models would also be an ideal tool for large-scale CKD risk stratification. However, as the equations for estimating glomerular filtration rate (eGFR), the CKD risk estimation models tend to be population-specific. Only a few of the existing CKD risk prediction algorithms have been validated in different populations across the world, and none of those has been tested in African populations [7]. Indeed, external validation is very important in the process of adopting prediction models in clinical or public health practice, as it enables their possible generalization [8].

To facilitate the adoption of CKD risk models as a risk stratification tool in African populations, we evaluated and compared the performance of two previously described prediction models for undiagnosed CKD [9, 10], in mixed-ancestry South Africans.

## Methods

### Study population and design

The Cape Town Bellville-South study cohort served as the basis for the model validations. Baseline assessments were conducted from 2008 to 2011, with standardized collection of information on medical history, cardio-metabolic risk factors and serum chemistries [11]. The study was approved by the Ethics Committee of the Cape Peninsula University of Technology, Faculty of Health and Wellness Sciences (CPUT/HW-REC 2008/002 and CPUT/HW-REC 2010). The study was conducted according to the Code of Ethics of the World Medical Association (Declaration of Helsinki). All participants signed written informed consent after all the procedures had been fully explained in the language of their choice.

### Identification of prediction models to validate

The models of interest, that predict prevalent undiagnosed CKD, were identified from a recent systematic review [7], with an update of the search in up to July 2014, to identify possible new models. We mainly focused on models developed using non-invasively measured variables, especially those available in the Bellville-South cohort. Two prevalent undiagnosed CKD prediction models developed from cross-sectional studies were selected; namely the Korean and Thai models [9, 10]. These models were developed on samples of 6565 participants aged 30 years or more for the Korean and 3459 participants aged 18 years or more for the Thai model. Both models defined CKD on the basis of eGFR using the Modification of Diet in Renal Disease (MDRD), Table 2. Of the two models, only the Korean one has been previously externally validated once [10].

### Outcomes

We used two definitions of kidney disease in accordance with the definitions applied in the original studies of models: eGFR <60 ml/min/1.73 m^2^ (only) and ‘any nephropathy’ including any of the stages I to V of the Kidney Disease: Improving Global Outcomes Chronic Kidney Disease (KDIGO) classification [3].

### Baseline assessments

Participants received a standardized interview and physical examination during which blood pressure was measured according to the World Health Organisation (WHO) guidelines [12] using a semi-automated digital blood pressure monitor (Rossmax PA, USA) on the right arm in the sitting position. Anthropometric measurements were performed three times and their average used for analysis: weight (kg), height (cm), waist (cm) and hip (cm) circumferences. Participants with no history of doctor diagnosed diabetes mellitus underwent a 75 g oral glucose tolerance test (OGTT) as recommended by the WHO [13]. Blood samples were obtained after an overnight fast for the assessment of glucose, glycated haemoglobin (HbA_1C_) certified by National Glycohaemoglobin Standardisation Programme (NGSP), creatinine (standardised assay), total cholesterol (TC), high density lipoprotein cholesterol (HDL-c), and triglycerides (TG). These parameters were determined by the Cobas 6000 Clinical Chemistry instrument (Roche Diagnostics, Germany). Low density lipoprotein cholesterol (LDL-c) was calculated using Friedewald formula [14]. Kidney function was defined using estimated glomerular filtration rate (eGFR) calculated using the four -variable Modification of Diet in Renal Disease (MDRD) equation [15, 16]. The MDRD equation was primarily used to define kidney function, consistent with what was done in the original models; however, we also considered the Chronic Kidney Disease Epidemiology Collaboration (CKD-EPI) equation for GFR estimation [17].

### Handling of missing data

Some of the predictors included in the tested models were not evaluated in the Bellville South study. These include anaemia (included in the Korean model) and kidney stones (component of the Thai model), which were consequently excluded from the validation. History of cardiovascular disease used as a predictor in the Korean model was inconsistently evaluated in our sample; hence the use of statin was considered as a proxy for cardiovascular disease. We opted to exclude participants with missing data on all other predictors, given the challenges of applying advanced imputation techniques for missing data in validation studies of risk models.

### Statistical methods

The CKD risk models of interest were validated in the overall sample, and then in subgroups, using the original formulas and with and without any recalibration. The predicted probability of undiagnosed CKD for each participant was estimated using the relevant predictors for each model (Table 1 & Additional file 1: Table S1) [9, 10]. Models’ performance was assessed through discrimination and calibration. Discrimination (ability of the model’s to distinguish those with prevalent undiagnosed CKD from those without the conditions) was assessed with C-statistic and non-parametric methods [18]. Calibration (agreement between the probability of the outcome of interest as estimated by the model, and the observed outcome frequencies) was assessed graphically by plotting the predicted risk against the observed outcome rate, supplemented with the Hosmer and Lemeshow goodness of fit test [19, 20]. The agreement between the expected (E) and observed (O) CKD rates (E/O) was assessed overall and within pre-specified groups of participants. The 95 % confidence intervals (CIs) for the expected/observed probabilities (E/O) ratio were calculated assuming a Poisson distribution [19]. We also calculated the Yates slope (difference between mean predicted probability of CKD for participants with and without prevalent CKD, with higher values indicating better performance) and the Brier score (squared difference between predicted probability and actual outcome for each participant with values ranging between 0 for a perfect prediction model and 1 for no match in prediction and outcome) [21, 22]. To determine optimal cut-off for maximising the potential effectiveness of a model, the Youden’s J statistic (Youden’s index) was used to determine the best threshold [23], with sensitivity and specificity determined for each threshold.

**Table 1:** Characteristics of participants by sex in the Bellville South cohort

Characteristics	Overall	Men	Women	P-value
N	902	211	691	
Age, years (SD)	55 (15)	56 (15)	53 (14)	0.068
Body mass index, kg/m^2^ (SD)	29.9 (7.2)	26.2 (6.2)	31.0 (7.1)	<0.0001
Waist circumference, cm (SD)	97 (15)	94 (15)	98 (15)	0.002
Hypertension, n (%)	449 (49.8)	104 (49.3)	346 (49.9)	0.871
Diabetes, n (%)	252 (27.9)	63 (29.8)	189 (27.3)	0.478
Statin use, n (%)	45 (5.0)	16 (7.6)	29 (4.2)	0.048
Smoking, n (%)	363 (40.2)	108 (51.2)	255 (36.9)	0.0002
Systolic blood pressure, mmHg (SD)	123 (19)	127 (18)	122 (19)	0.004
Diastolic blood pressure, mmHg (SD)	75 (12)	76 (12)	74 (13)	0.041
Height, m (SD)	1.59 (0.09)	1.68 (0.08)	1.56 (0.07)	<0.0001
Fasting blood glucose, mmol/L (SD)	6.4 (3.1)	6.6 (3.8)	6.4 (2.8)	0.355
HbA1c, % (SD)	6.3 (1.4)	6.4 (1.7)	6.2 (1.3)	0.307
Total cholesterol, mmol/L (SD)	5.6 (1.2)	5.3 (1.1)	5.7 (1.2)	<0.0001
High density lipoprotein cholesterol, mmol/L (SD)	1.3 (0.3)	1.2 (0.3)	1.3 (0.3)	0.0009
Weight, kg (SD)	75 (18)	74 (17)	75 (18)	0.175
Triglyceride, mmol/L (SD)	1.5 (0.9)	1.5 (0.9)	1.5 (0.9)	0.543
Creatinine, μmol/l (SD)	83 (20)	94 (20)	80 (19)	<0.0001
Median urinary albumin/creatinine (ACR), mg/mmol [25^th^-75^th^ percentiles]	0.73 [0.41-1.56]	0.67 [0.32-1.82]	0.75 [0.44-1.50]	0.126
Albuminuria (ACR > =30), n (%)	21 (2.3)	4 (1.2)	17 (2.5)	0.634
Estimated glomerular filtration rate (eGFR, MDRD), ml/min/1.73 m^2^ (SD)	71.5 (19.4)	76.6 (20.2)	69.9 (18.8)	<0.0001
Chronic kidney disease (CKD, eGFR (MDRD) < 60), n (%)	259 (28.7)	42 (19.9)	217 (31.4)	0.001
CKD (eGFR (MDRD) < 60 and/or albuminuria), n (%)	268 (29.7)	42 (19.9)	226 (32.7)	0.0004
eGFR (CKD-EPI), ml/min/1.73 m^2^ (SD)	77.5 (20.6)	80.7 (19.9)	76.6 (20.8)	0.011
Chronic kidney disease (CKD, eGFR (CKD-EPI) < 60), n (%)	186 (20.6)	35 (16.6)	151 (21.8)	0.098
CKD (eGFR (CKD-EPI) < 60 and/or albuminuria), n (%)	196 (21.7)	35 (16.6)	161 (23.3)	0.038

CKD-EPI, Chronic Kidney Disease Epidemiology Collaboration; MDRD, Modification of Diet in Renal Disease; SD, standard deviation

To minimize differences in CKD prevalence between the development and test populations, and thus improve performance, models were recalibrated to the test-population-specific CKD prevalence using intercept adjustment [24]. The calculated correction factor is based on the mean predicted risk and the prevalence in the validation set and is the natural logarithm of the odds ratio of the mean observed prevalence and the mean predicted risk [24]. The main analysis focused on the overall cohort, and subgroups analyses were by sex, age (<60 vs. ≥60 years) and BMI (<25 kg/m^2^ vs. ≥25 g/m^2^). Additionally, we conducted sensitivity analyses, to assess all the aforementioned aspects of model using the CKD-EPI equation [17] to estimate kidney function and define CKD.

For all analyses, we used the statistical software R Version 3.0.3 [2014-03-04] (The R Foundation for statistical computing, Vienna, Austria). A p-value <0.05 was used to characterize statistically significant results.

## Results

### Participants’ characteristics

Of the 1285 participants screened in the Bellville South cohort, 383 were excluded because of missing data on predictor variables or renal function. Therefore, the final analytic sample included 902 participants. The excluded participants (Additional file 1: Table S2) were more likely to be men, younger, taller and to have hypertension, higher systolic or diastolic blood pressure, lower total cholesterol or serum creatinine, but a higher eGFR.

The characteristics of study participants stratified by sex are presented in Table 1. Overall, compared to women, men were less obese (both by BMI and abdominal circumference), but had lower total cholesterol, HDL cholesterol, and lower prevalence of CKD defined as eGFR < 60 ml/min/1.73 m^2^ or as eGFR <60 ml/min/1.73 m^2^ and/or albuminuria. Systolic or diastolic blood pressure, height, serum creatinine, eGFR and proportion of smokers were significantly higher in men than in women.

### Prediction of prevalent undiagnosed CKD in the overall sample

**Fig. 1:**
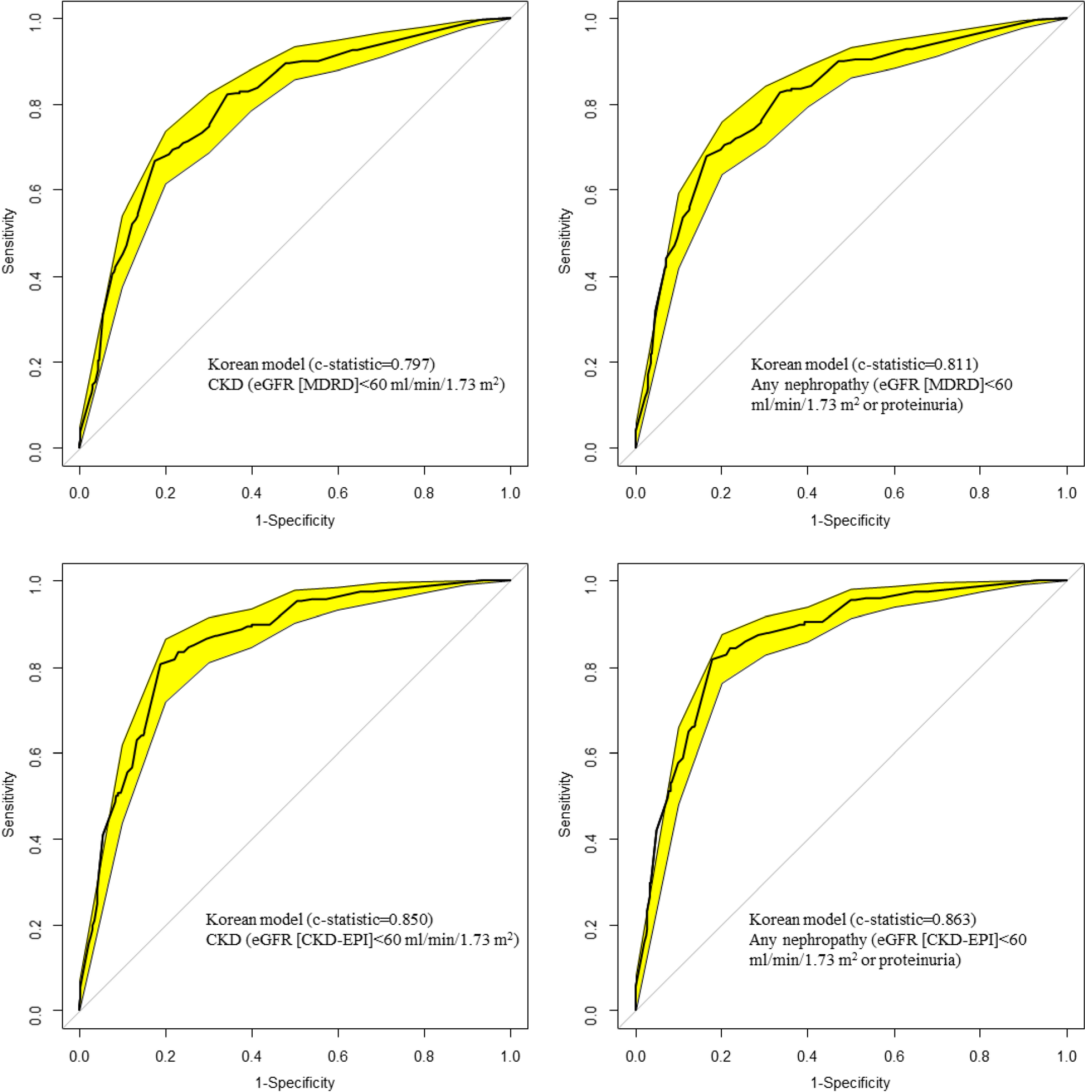
Receiver operating characteristic curves (ROC) showing the discrimination of the Korean model. The yellow band around curve represents the 95 % confidence interval; the diagonal line at 45° is the line of no discrimination. Figure panels are for the outcome of CKD (eGFR < 60 ml/min/1.73 m^2^) for the left panels and ‘any nephropathy (eGFR < 60 ml/min/1.73 m^2^ or proteinuria) for the right panels, and for MDRD defined CKD (upper panels) and CKD-EPI defined CKD (lower panels)

**Fig. 2:**
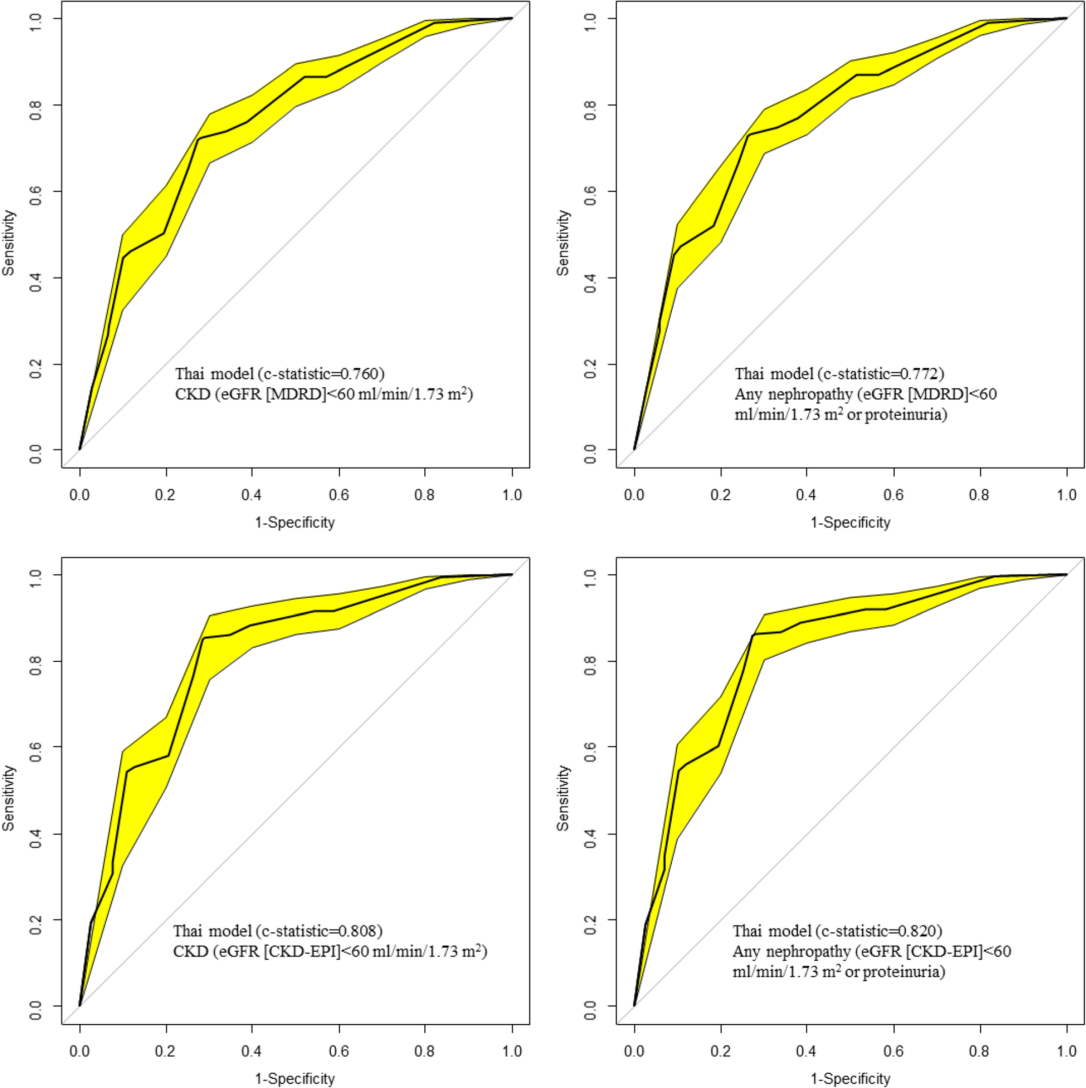
Receiver operating characteristic curves (ROC) showing the discrimination of the Thai model. The yellow band around curve represents the 95 % confidence interval; the diagonal line at 45° is the line of no discrimination. Figure panels are for the outcome of CKD (eGFR < 60 ml/min/1.73 m^2^) for the left panels and ‘any nephropathy (eGFR < 60 ml/min/1.73 m^2^ or proteinuria) for the right panels, and for MDRD defined CKD (upper panels) and CKD-EPI defined CKD (lower panels)

A total of 259 participants (28.7 %) had undiagnosed CKD (eGFR < 60 ml/min/1.73 m^2^). When using the eGFR < 60 ml/min/1.73 m^2^ and/or albuminuria as the definition of CKD, nine additional patients (solely women) would have the condition (Table 1). Table 2 and Figs. 1 and 2 show the discriminative ability of two prediction models selected. The C-statistic was 0.760 (95 % CI: 0.726-0793) for the Korean model for predicting ‘eGFR < 60 ml/min/1.73 m^2’^ and 0.811 (0.780-0.842) for ‘any nephropathy’; corresponding figures were 0.797 (0.765-0.829) and 0772 (0.739-0.805) for the Thai model. In all pairwise comparisons, the Korean model always had significantly better discrimination than the Thai model (all p < 0.0001).

**Table 2:** Overview of the tested models of prevalent chronic kidney disease (CKD) prediction and their performance for the original model and the intercept adjusted model, based on MDRD equation defined CKD

Characteristics	Korean risk score [10]				Thai risk score [9]				Bellville South
Authors	Kwon *et al.*				Thakkinstian *et al.*				
Year published	2011				2011				
Country	South Korea				Thailand				
Validation	Internal, External				Internal				
Sample size	6565				3459				
Design	Cross-sectional				Cross-sectional				
Age range	≥30 years				≥18 years				
Population	Korean				Thai				
Definition of of CKD	eGFR (MDRD) ≤ 60 ml/min per 1.73 m^2^				stage I-V based on eGFR (MDRD)				
Development c-statistic	0.83				0.77				
Predictors									
*Age*	Yes				Yes				Yes
*Sex*	Yes				No				Yes
*Anemia*	Yes				No				No
*Hypertension*	Yes				Yes				Yes
*Diabetes*	Yes				Yes				Yes
*History of CVD*	Yes				No				No (statin use)
*Proteinuria*	Yes				No				Yes
*Kidney stone*	No				Yes				No
Performance	Original		Adjusted		Original		Adjusted		
Outcome	eGFR < 60	Any CKD	eGFR < 60	Any CKD	eGFR < 60	Any CKD	eGFR < 60	Any CKD	
E/O (95 % CI)	0.07 (0.07-0.08)	0.07 (0.06-0.08)	0.76 (0.67-0.86)	0.76 (0.67-0.85)	0.90 (0.79-1.01)	0.87 (0.77-0.98)	0.98 (0.87-1.10)	0.97 (0.86-1.09)	
Brier score	0.265	0.274	0.164	0.161	0.166	0.166	0.165	0.164	
Yates slope	0.026	0.029	0.208	0.225	0.191	0.199	0.200	0.211	
C-statistic (95 % CI)	0.797 (0.765-0.829)	0.811 (0.780-0.842)	0.797 (0.765-0.829)	0.811 (0.780-0.842)	0.760 (0.726-0.793)	0.772 (0.739-0.805)	0.760 (0.726-0.793)	0.772 (0.739-0.805)	
Best threshold	0.03	0.02	0.30	0.31	0.27	0.27	0.31	0.32	
Sensitivity (%)	82	84	82	84	73	74	73	74	
Specificity (%)	67	68	67	68	72	73	72	73	

CI: confidence interval, CKD; chronic kidney disease, CVD: cardiovascular disease, E/O: expected/observed

**Fig. 3:**
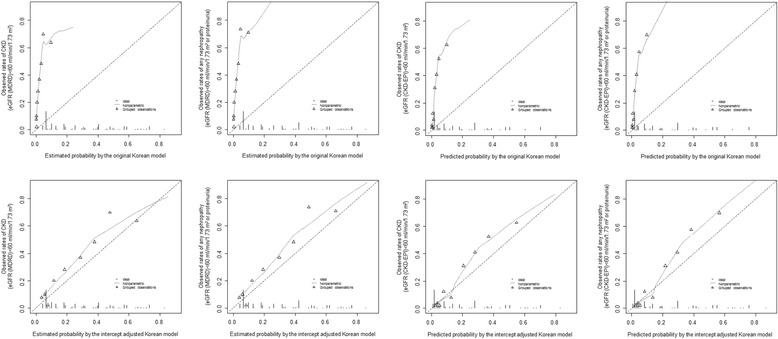
Calibration curves for the Korean model before (upper panels) and after intercept adjustment (lower panels), for the outcome of CKD (eGFR < 60 ml/min/1.73 m^2^) for the first and third column and ‘any nephropathy’ (eGFR < 60 ml/min/1.73 m^2^ or proteinuria) for the second and left columns. For each figure panel the broken diagonal line at 45° represents the ideal calibration. Participants are grouped into percentiles across increasing estimated probability. The vertical lines at the bottom of the graph depict the frequency distribution of the calibrated probabilities. eGFR is from MDRD equation (1^st^ and 2^nd^ columns) and CKD-EPI equation (3^rd^ and 4^th^ columns)

**Fig. 4:**
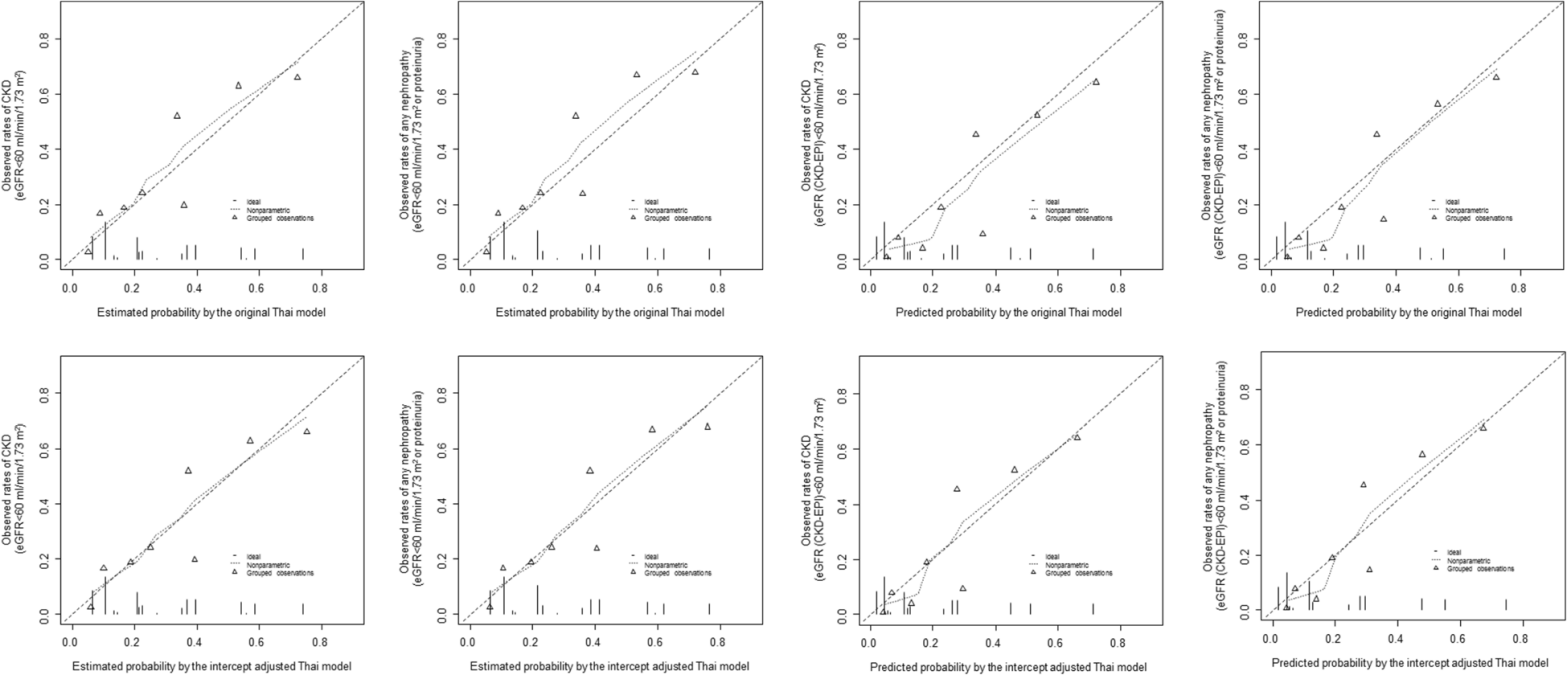
Calibration curves for the Thai model before (upper panels) and after intercept adjustment (lower panels), for the outcome of CKD (eGFR < 60 ml/min/1.73 m^2^) for the first and third column and ‘any nephropathy’ (eGFR < 60 ml/min/1.73 m^2^ or proteinuria) for the second and left columns. For each figure panel the broken diagonal line at 45° represents the ideal calibration. Participants are grouped into percentiles across increasing estimated probability. The vertical lines at the bottom of the graph depict the frequency distribution of the calibrated probabilities. eGFR is from MDRD equation (1^st^ and 2^nd^ columns) and CKD-EPI equation (3^rd^ and 4^th^ columns)

CKD was slightly under estimated by the Thai model by 10 % (95 % CI: 1-21 %) and 13 % (2 %-23 %) for ‘eGFR < 60 ml/min/1.73 m^2^’ and ‘any nephropathy’ respectively. However, it was largely underestimated by the Korean model by 93 % (92-93 %) for ‘eGFR < 60 ml/min/1.73 m^2^’ and ‘any nephropathy’ (Table 2). The calibration curves are shown in Figs. 3 and 4. The curves were steeper for the Korean model and always above the diagonal line of perfect calibration, indicating a systematic risk underestimation. With the Thai model, the curve was parallel to and always above the diagonal line. It was mostly closer to this line in lower risk strata than in the upper ones, suggesting a selective risk underestimation among participants at high risk. The Yates slope and Brier score are also presented in Table 2.

### Prediction of prevalent undiagnosed CKD in subgroups

The C-statistic varied widely across complementary subgroups (Table 3). The two models had a better discrimination among men than women, with a C-statistic of 0.856 (0.792-0.920) for the Korean model, and 0.834 (0.770-0.899) for the Thai model, irrespective of the CKD definition used. The C-statistic was lower in younger participants (age <60 years), with values of 0.678 (0.615-0.741) and 0.689 (0.626-0.751) for the ‘eGFR <60 ml/min/1.73 m^2^’ and ‘any nephropathy’ respectively for the Korean model, and 0.608 (0.550-0.666) and 0.619 (0.561-0.676) for the Thai model. The two models showed an improved discrimination of CKD in lean participants (BMI <25 kg/m^2^) than in overweight and obese participants (BMI ≥25 kg/m^2^).

**Table 3:** Discrimination and calibration statistics for chronic kidney diseases risk model performance in subgroups of participants by gender, age and body mass index (BMI); for the outcome of CKD based on MDRD equation predicted glomerular filtration rate with or without proteinuria

Models (outcome)		Men	Women	Age < 60 years	Age ≥ 60 years	BMI < 25 kg/m^2^	BMI ≥ 25 kg/m^2^
Thai model (eGFR < 60 ml/min/1.73 m^2^)	E/O (95 % CI)	1.49 (1.10-2.02)	0.88 (0.77-1.00)	1.13 (0.90-1.42)	0.91 (0.79-1.05)	0.98 (0.76-1.26)	0.98 (0.85-1.12)
	Brier score	0.127	0.177	0.121	0.238	0.128	0.180
	Yates slope	0.267	0.190	0.0.032	0.083	0.259	0.175
	C-statistic (95 % CI)	0.834 (0.770-0.899)	0.752 (0.714-0.790)	0.608 (0.550-0.666)	0.626 (0.567-0.685	0.854 (0.802-0.906)	0.723 (0.681-0.765)
Korean model (eGFR < 60 ml/min/1.73 m^2^)	E/O (95 % CI)	0.99 (0.73-1.34)	0.72 (0.63-0.82)	0.68 (0.54-0.85)	0.80 (0.69-0.92)	0.75 (0.59-0.97)	0.76 (0.66-0.88)
	Brier score	0.112	0.179	0.116	0.243	0.129	0.177
	Yates slope	0.246	0.199	0.049	0.087	0.259	0.186
	C-statistic (95 % CI)	0.856 (0.792-0.920)	0.784 (0.747-0.821)	0.678 (0.615-0.741)	0.668 (0.610-0.726)	0.869 (0.814-0.923)	0.766 (0.727-0.806)
Thai model (eGFR < 60 ml/min/1.73 m^2^ or proteinuria)	E/O (95 % CI)	1.53 (1.13-2.08)	0.87 (0.76-0.99)	1.15 (0.92-1.43)	0.89 (0.78-1.03)	0.96 (0.75-1.23)	0.97 (0.85-1.12)
	Brier score	0.127	0.175	0.123	0.233	0.125	0.179
	Yates slope	0.267	0.205	0.040	0.092	0.276	0.184
	C-statistic (95 % CI)	0.834 (0.770-0.899)	0.768 (0.732-0.805)	0.619 (0.561-0.676)	0.646 (0.587-0.704)	0.870 (0.821-0.919)	0.734 (0.693-0.775)
Korean mdel (eGFR < 60 ml/min/1.73 m^2^ or proteinuria)	E/O (95 % CI)	1.02 (0.75-1.38)	0.71 (0.62-0.80)	0.69 (0.55-0.86)	0.78 (0.68-0.91)	0.74 (0.58-0.95)	0.76 (0.66-0.87)
	Brier score	0.112	0.176	0.116	0.235	0.125	0.175
	Yates slope	0.246	0.219	0.059	0.104	0.284	0.201
	C-statistic (95 % CI)	0.856 (0.792-0.920)	0.801 (0.766-0.836)	0.689 (0.626-0.751)	0.696 (0.640-0.753)	0.886 (0.834-0.937)	0.778 (0.740-0.817)

eGFR, estimated glomerular filtration rate; E/O, Expected/Observed event rate; 95 % CI: 95 % confidence interval

The overall calibration as expressed by the E/O ratio varied across subgroups depending on the model. Both models overestimated CKD risk in men and underestimate it in women. The Thai model (‘eGFR <60 ml/min/1.73 m^2^’ and ‘any nephropathy’) overestimates CKD risk among younger participants, and both models underestimate the risk among older participants. The Thai model overestimates the risk of CKD in the BMI subgroups (Table 3).

### Recalibration through intercept adjustment

After recalibration through intercept adjustment, there was an almost perfect risk estimation by the Thai model for both CKD outcomes, with E/O of 0.98 (0.87-1.10) for ‘eGFR < 60 ml/min/1.73 m^2^’ and 0.97 (0.86-1.09) for ‘any nephropathy’ (Fig. 3), and calibration curve mostly following the diagonal line of perfect calibration (Fig. 4). However, the Korean model still slightly underestimated CKD risk by 24 % (33–15) for both outcomes. The Hosmer-Lemeshow test indicated a disagreement between the predicted and observed prevalence of undiagnosed CKD after intercept adjustment (all p < 0.0001), although there was attenuation in comparison with the original model (Additional file 1: Table S3).

### Optimal threshold for defining high risk of CKD

The optimal threshold for the intercept adjusted Thai model was 0.31 and 0.32 respectively for ‘eGFR < 60 ml/min/1.73 m^2^’ and for ‘any nephropathy’. Corresponding performance measures were 73 % and 74 % for sensitivity, and 72 % and 73 % for specificity. Optimal thresholds for the intercept adjusted Korean model were 0.30 for ‘eGFR < 60 ml/min/1.73 m^2^’ and 0.31 for ‘any nephropathy’. Accompanying performance measures were 82 % and 84 % for sensitivity, and 67 % and 68 % for specificity (Table 2).

### Sensitivity analysis – models validation for chronic kidney disease defined by CKD-EPI equation predicted glomerular filtration rate

The results obtained with the CKD-EPI equation were broadly similar to the one obtained using the CKD-MDRD equation to define CKD. Table 4 and Figs. 1 and 2 show the discriminative ability of the prediction models using the CKD-EPI equation. The C-statistic was 0.850 (0.821-0.880) for the Korean model for predicting ‘eGFR < 60 ml/min/1.73 m^2’^ and 0.863 (0.835-0.891) for ‘any nephropathy’; corresponding figures were 0.808 (0.775-0.842) and 0.820 (0.788-0.852) for the Thai model. Direct comparisons of the C-statistics indicated significant difference between the Thai and the Korean models (p < 0.0001 for CKD and p < 0.0001 for any CKD).

**Table 4:** Performance for the original model and the intercept adjusted model in the overall population based on CKD-EPI equation defined chronic kidney disease

Performance	Korean risk score [10]				Thai risk score [9]			
Model	Original	Adjusted	Original	Adjusted				
Outcome	eGFR < 60	Any CKD	eGFR < 60	Any CKD	eGFR < 60	Any CKD	eGFR < 60	Any CKD
E/O (95 % CI)	0.10 (0.09-0.12)	0.10 (0.09-0.11)	0.80 (0.69-0.92)	0.79 (0.69-0.91)	1.25 (1.08-1.44)	1.18 (1.03-1.38)	1.04 (0.91-1.21)	1.03 (0.90-1.19)
Brier score	0.187	0.197	0.123	0.123	0.128	0.128	0.126	0.127
Yates slope	0.034	0.036	0.212	0.230	0.236	0.243	0.213	0.225
C-statistic (95 % CI)	0.850 (0.821-0.880)	0.863 (0.835-0.891)	0.850 (0.821-0.880)	0.863 (0.835-0.891)	0.808 (0.775-0.842)	0.820 (0.788-0.852)	0.808 (0.775-0.842)	0.820 (0.788-0.852)
Best threshold	0.02	0.02	0.22	0.22	0.25	0.27	0.22	0.23
Sensitivity (%)	81	82	81	82	71	72	71	72
Specificity (%)	82	82	82	82	85	86	85	86

CKD; chronic kidney disease, eGFR, estimated glomerular filtration rate; E/O, Expected/Observed event rate; 95 % CI: 95 % confidence interval

CKD was slightly overestimated by the original Thai model and broadly underestimated by the original Korean model, as indicated by E/O ratio (Table 4). The calibration curves for the Thai and Korean models (Figs. 3 and 4), as well as the Yates slope and Brier score (Table 4) indicated systematic risk overestimation and underestimation, respectively. As for prediction in subgroups (Table 5), the two models had a better discrimination (C-statistic) among men than women, in older participants (age <60 years) compared to younger ones, and in lean participants (BMI <25 kg/m^2^) than in overweight and obese participants (BMI ≥25 kg/m^2^). The Thai models overestimated CKD risk in men but overestimates the risk of CKD in the obese BMI subgroups; and the Korean model tended to overestimate risk in men (Table 5).

**Table 5:** Discrimination and calibration statistics for chronic kidney diseases (CKD) risk model performance in subgroups of participants by gender, age and body mass index (BMI), for the outcome of CKD based on CKD-EPI equation predicted glomerular filtration rate with or without proteinuria

Models (outcome)		Men	Women	Age < 60 years	Age ≥ 60 years	BMI < 25 kg/m^2^	BMI ≥ 25 kg/m^2^
Thai model (eGFR < 60 ml/min/1.73 m^2^)	E/O (95 % CI)	1.38 (0.99-1.92)	0.97 (0.82-1.13)	2.14 (1.49-3.08)	0.84 (0.72-0.99)	0.96 (0.72-1.28)	1.08 (0.91-1.27)
	Brier score	0.105	0.132	0.056	0.242	0.106	0.133
	Yates slope	0.254	0.206	0.019	0.076	0.246	0.199
	C-statistic (95 % CI)	0.850 (0.787-0.912)	0.802 (0.764-0.840)	0.573 (0.479-0.667)	0.614 (0.556-0.673)	0.872 (0.820-0.923)	0.784 (0.742-0.826)
Korean model (eGFR < 60 ml/min/1.73 m^2^)	E/O (95 % CI)	0.88 (0.63-1.23)	0.78 (0.82-1.13)	1.25 (0.87-1.80)	0.72 (0.61-0.84)	0.73 (0.55-0.98)	0.83 (0.91-1.27)
	Brier score	0.098	0.131	0.049	0.247	0.110	0.128
	Yates slope	0.221	0.208	0.031	0.082	0.231	0.203
	C-statistic (95 % CI)	0.887 (0.835-0.939)	0.842 (0.807-0.876)	0.676 (0.587-0.766)	0.674 (0.617-0.730)	0.891 (0.844-0.937)	0.833 (0.797-0.870)
Thai model (eGFR < 60 ml/min/1.73 m^2^ or proteinuria)	E/O (95 % CI)	1.44 (1.03-2.01)	0.95 (0.81-1.10)	2.05 (1.45-2.90)	0.84 (0.97-0.72)	0.95 (0.72-1.25)	0.82 (0.70-0.97)
	Brier score	0.106	0.133	0.059	0.240	0.106	0.135
	Yates slope	0.259	0.220	0.038	0.085	0.264	0.208
	C-statistic (95 % CI)	0.850 (0.787-0.912)	0.817 (0.781-0.853)	0.611 (0.517-0.705)	0.632 (0.574-0.690)	0.887 (0.839-0.935)	0.793 (0.753-0.834)
Korean mdel (eGFR < 60 ml/min/1.73 m^2^ or proteinuria)	E/O (95 % CI)	0.92 (0.66-1.29)	0.77 (0.66-0.89)	1.20 (0.85-1.70)	0.71 (0.61-0.83)	0.72 (0.55-0.95)	0.82 (0.70-0.97)
	Brier score	0.097	0.130	0.051	0.241	0.108	0.128
	Yates slope	0.229	0.230	0.055	0.100	0.257	0.218
	C-statistic (95 % CI)	0.887 (0.835-0.939)	0.858 (0.825-0.890)	0.709 (0.622-0.797)	0.700 (0.645-0.755)	0.907 (0.864-0.950)	0.844 (0.808-0.880)

eGFR, estimated glomerular filtration rate; E/O, Expected/Observed event rate; 95 % CI: 95 % confidence interval

After recalibration through intercept adjustment, CKD risk was estimated better by both models for both CKD outcomes, but more so for the Thai model that the Korean one. The Hosmer-Lemeshow test indicated disagreement between the predicted and observed prevalence of undiagnosed CKD (all p < 0.0001) though there was attenuation in comparison with the original model (Additional file 1: Table S3).

## Discussion

To our knowledge, our study is the first conducted in Sub-Saharan Africa on CKD risk prediction models. In the South African cohort of Bellville South, the two models had a good-to-acceptable discrimination for the presence of undiagnosed CKD, with a better performance in older age group, men and normal weight participants. Calibration was also acceptable for the Thai model while substantial risk underestimation was observed with the Korean model, with however improvement after recalibration through intercept adjustment. At the optimal threshold derived from our sample, both models had good sensitivity and acceptable specificity to select participants who are more likely to be diagnosed with CKD via biological tests. The performance of the models was not significantly influenced by the methods used to defined impaired kidney function, CKD-MDRD or CKD-EPI equations. Interestingly, both models are based on non-invasively measurable predictors in routine clinical and community-based settings. Altogether, our study suggests that, with little additional efforts models developed in Asians to screen the risk of prevalent undiagnosed CKD, can be adapted to accurately serve the same purpose in African populations; therefore obviating the need to develop new models from scratch in the African settings.

### Comparison with other external validation studies

Compared to other external validation studies of CKD risk models, we found the highest discrimination values [7]. We also used multiple metrics of performance assessment compared to existing validations studies. Furthermore, the same group of investigators who developed original models has mainly conducted the extant validation studies. This tends to be methodologically inferior and quantitatively insufficient to provide good indicators of models’ behavior in various populations. The variation in model performance across subgroups in our study may simply reflect differences in the distribution of the disease and its risk factors. For instance, overestimation observed among of males and youngsters in our population may simply reflect the predominance of these groups in our population. Also, that the discrimination ability of models is better in older participant is unsurprising as CKD occurrence is strongly related to aging [1].

### Implications and uses of CKD risk models in the African context

A recent overview of CKD studies conducted in Africa has reported a pooled CKD prevalence of 13.9 %, with no difference between urban and rural studies [25]. Those seen with CKD in community based studies in Africa are more likely to be people not previously diagnosed with the condition; in line with the poor detection rates already reported in Africa for major non-communicable diseases such as hypertension [26] and diabetes mellitus [27]. This reflects the lack of or the insufficient ongoing effort to screen people for CKD and common NCDs in this setting. Furthermore, hospital-based studies have reported unacceptable rates of patients referrals with CKD to nephrologists, usually at the terminal stage of the disease, including even among patients receiving ongoing care from other non-nephrologist physicians [28]. The scope of needs and challenges in term of CKD risk screening and prevention in Africa therefore is broad and invites both health facilities based and community based actions.

The investigated risk models have potential applications in the prevention and management of CKD in Africa. Indeed, CKD is a silent disease that usually presents at the ESRD stage with limited chances of survival. Though the tested models have not been investigated for improvement of outcomes in routine practice, their performances indicate that these could be used to boost the detection of CKD in Africa both in clinical practice and at a larger scale in low-income settings. Practitioners can use the derived risk from these tools to detect CKD followed by timely referral to a renal physician, and make recommendations on behavioral changes in high–risk patients who are ultimately not found to have the condition. Indeed, communication of risks to patient using valid tools may motivate them to adhere to healthy habits and prescribed therapies. Using these models, clinicians may be able to increase the frequency of monitoring to individual risk. Considering the prohibitive costs of renal replacement therapy in this environment, early detection and/or prevention of progression of CKD are an imperative for countries in this region. However, how to best achieve this is still unclear, especially in low-income setting. Indeed, easy to use and inexpensive tools may be useful as the numbers of and complexity of predictors and cost of measurement would limit applicability in various settings.

### Strengths and limitations

Our study has strengths including a community-based sample, and the rigorous and detailed external validation approaches. However, the limitations of this study need to be mentioned. The approaches used to account for predictors that were completely missing (e.g., proxy variables, predictor omission) could decrease discrimination of the models. It is well known that deletion of participants with missing values (frequent in large studies) leads to biased results [18]. Our sample was limited in terms of the race/ethnicity variability, thus our results might not be generalizable to all African countries or ethnic groups. The definition of CKD was based on the MDRD equation. The MDRD equation may provide less accurate estimates of GFR, compared with estimates derived from the more recent CKD-EPI equation. Our sensitivity analyses however suggest that the models’ performance was equally acceptable-to-good regardless of the kidney function estimator used. Also, more risk predictions tools could have been tested in our population, but this was not done due to the lack of necessary information on key variables.

## Conclusion

Our study has highlighted the acceptable performance of CKD risk models developed in Asian population when applied to an African population. Given the strong need for reliable and convenient tool for identifying undiagnosed or predicting future CKD in a cost-effective manner, especially low-income settings like Africa where CKD prevalence is galloping particular, the tested models can be effectively used in a stepwise approach to identifying people with undiagnosed CKD. However, the assessment of the impact of risk model use on patient outcomes is needed before it being incorporated into routine clinical practice guidelines.

## Additional file

Additional file 1:
**Validation of two prediction models of undiagnosed chronic kidney disease in mixed-ancestry South Africans.**

## Abbreviations

BMI: Body mass index
CI: Confidence interval
CKD: Chronic kidney disease
CVD: Cardiovascular disease
E/O: Expected/observed probability
eGFR: estimated glomerular filtration rate
ERSD: End-stage of renal disease
HbA1c: Glycated haemoglobin
HDL-c: High density lipoprotein cholesterol
KDIGO: Kidney Disease: Improving Global Outcomes Chronic Kidney Disease
KDOQI: Kidney Disease Outcomes Quality Initiative
LDL-c: Low density lipoprotein cholesterol
MDRD: Modification of Diet in Renal Disease
NGSP: National Glycohaemoglobin Standardisation Programme
OGTT: Oral glucose tolerance test
TC: Total cholesterol
TG: Triglycerides
WHO: World Health Organisation
